# Wetting and evaporation behavior of dilute sodium dodecyl sulfate droplets on soft substrates under a direct current electric field

**DOI:** 10.1038/s41598-024-58166-9

**Published:** 2024-03-27

**Authors:** Biao Jiang, Shuai Xu, Yingfa Lu, Yingsong Yu

**Affiliations:** 1 Department of Mechanics, School of Civil Engineering, Architecture and Environment, Hubei University of Technology, Wuhan, 430068 China; 2 Key Laboratory of Intelligent Health Perception and Ecological Restoration of Rivers and Lakes, Ministry of Education, Hubei University of Technology, Wuhan, 430068 China; 3 Innovation Demonstration Base of Ecological Environment Geotechnical and Ecological Restoration of Rivers and Lakes, Hubei University of Technology, Wuhan, 430068 China

**Keywords:** Engineering, Physics

## Abstract

Wetting and evaporation behavior of dilute sodium dodecyl sulfate (SDS) droplets on planar polydimethylsiloxane (PDMS) surfaces under a direct current (DC) electric field were experimentally investigated. Two characteristic voltages—actuation voltage and saturation voltage were observed in the electrowetting of dilute SDS droplets on PDMS surfaces. It was found that for dilute SDS droplets with a fixed SDS concentration substrate elasticity has an obvious influence on actuation voltage, and saturation voltage increased with the increase of mass ratio of PDMS surfaces. SDS concentration was also found to obviously influence actuation voltage and saturation voltage when SDS concentration was in a certain range. For the case of evaporation of sessile dilute SDS droplets on PDMS surfaces with the application of a DC electric field, substrate elasticity, SDS concentration and the magnitude of applied voltage were all found to have an influence on the duration of CCR stage. Moreover, contact angle hysteresis for dilute SDS droplets on a planar PDMS 10:1 surface under different applied voltage was measured and it was found that the magnitude of applied voltage greatly influenced contact angle hysteresis, which also depends on SDS concentration and KCl concentration.

## Introduction

There are a wide variety of applications for evaporation of sessile droplets, including inkjet printing, micro/nanofluidics, and DNA/RNA mapping^1–6^. The addition of surfactants and the application of an electric field make the evaporation of sessile droplets more complex, since surfactant molecules are adsorbable at the liquid–vapor and solid–liquid interfaces^7–11^ and desorbed at the solid–liquid interface under an electric field^12^. Hence, it is crucial to investigate the wetting and evaporation behavior of small droplets containing a small quantity of surfactant.

The evaporation of sessile droplets normally occurs in three stages sequentially, namely, the constant contact radius (CCR) stage, constant contact angle (CCA) stage, and mixed evaporation stage, which were first proposed by Picknett and Bexon^13^ in 1977. Whether the CCR stage dominates the evaporation greatly depends on contact angle hysteresis (CAH)^14,15^. In contrast to hard substrates, soft substrates have a very low Young’s modulus, usually in the range of several MPa to several tens of kPa. Consequently, when a minor droplet is gently deposited on a soft substrate, there will be unneglected surface deformation in the substrate because of the vertical component of the liquid–vapor interfacial tension^16–18^, which is not balanced. The deformation of the substrate contributes to CAH, resulting in an extended CCR stage. Therefore, it is urgent to investigate the evaporation of sessile droplets on soft substrates.

During the latest three decades, electrowetting on dielectrics (EWOD) has shown great advances in its accuracy and precision in manipulating small droplets such as coalescence, splitting, and directional transport and it has been successfully used in many fields such as micro-/nano-fluidics^19–22^. During EWOD, there are two characteristic electric voltages, actuation voltage and saturation voltage. The existence of actuation voltage is widely accepted to be caused by CAH^23^. Many studies suggest that the saturation voltage originates from charge trapping^24^, but there is no unified understanding of its origin. In the case of a voltage *V* varying between the actuation voltage and saturation voltage, the classical Young-Lippmann equation is valid^25^.

A surfactant can greatly decrease the surface tension of a liquid when its concentration is below the critical micelle concentration (CMC), above which the surface tension cannot be reduced any further^26^. Additionally, salts such as KCl and NaCl can reduce the CMC value of surfactant^27^. In the presence of an electric field, how will sessile droplets containing small amounts of surfactant and salts behave on soft substrates? What effect does substrate elasticity, concentration of surfactant and applied voltage have on droplet evaporation characteristics?

The present study investigated the wetting and evaporation behavior of dilute sodium dodecyl sulfate (SDS) droplets on planar polydimethylsiloxane (PDMS) surfaces with different mass ratios, while subjecting them to a direct current (DC) electric field. Electrowetting characteristics of these droplets were analyzed in relation to substrate elasticity and SDS concentration. The combined effect of SDS concentration and KCl concentration on CAH for dilute SDS droplets on PDMS surfaces without and with the application of a DC electric field was investigated. For the case of evaporation of dilute SDS droplets on planar PDMS surfaces with the presence of a DC electric field, it was found that substrate elasticity, SDS concentration and the magnitude of applied voltage all influenced the duration of CCR stage.

## Experiments and materials

**Figure 1:**
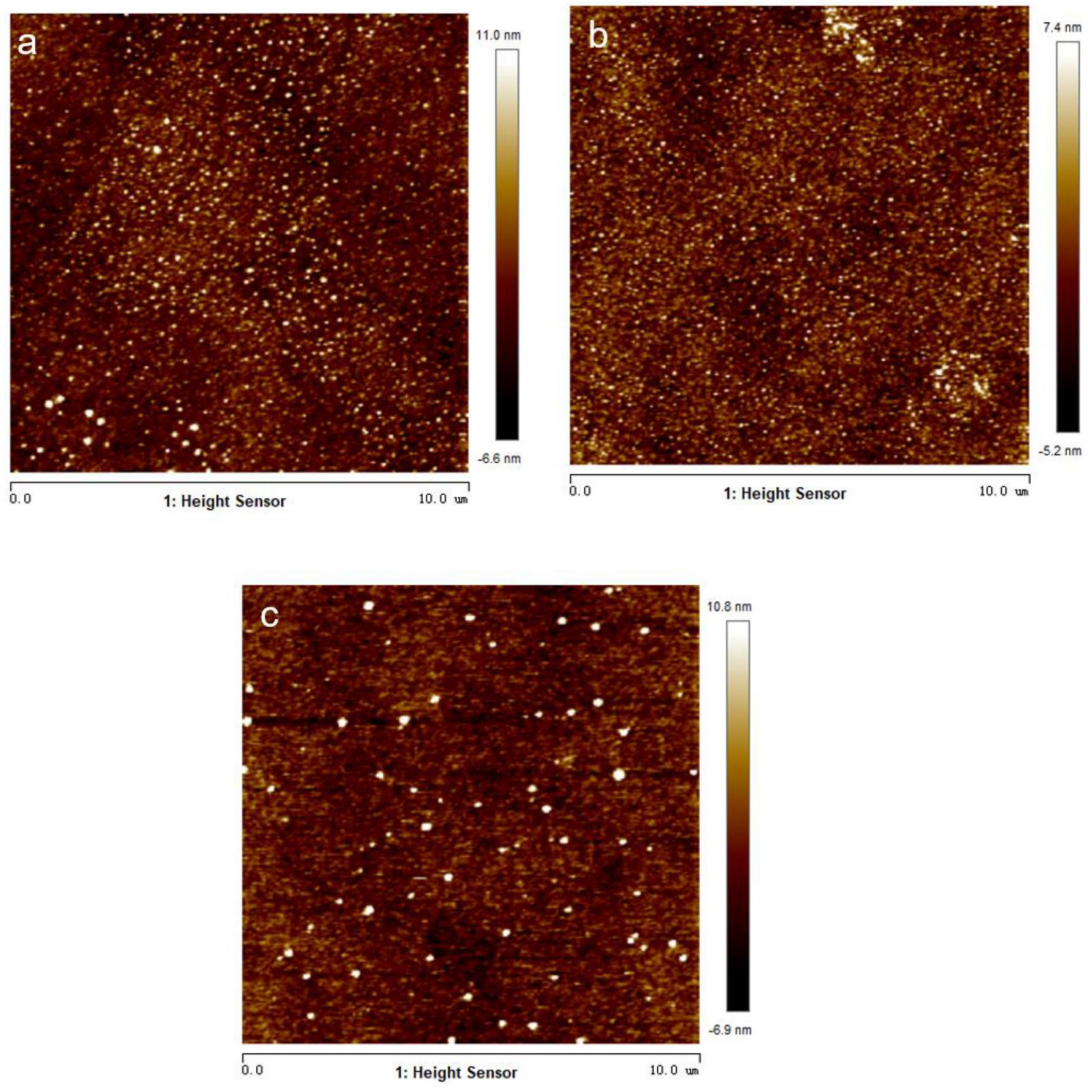
AFM images of PDMS surfaces. (**a**) PDMS 5:1, (**b**) PDMS 10:1, (**c**) PDMS 20:1.

Using the peeling-off method, PDMS surfaces were prepared by mixing silicone elastomer base (Sylgard 184) with curing agent (Sylgard 184 silicone elastomer curing agent) at the mass ratios of 5:1, 10:1 and 20:1, respectively, degassing the mixtures for 30 min and heating them at 90 ℃ for 4 h^28,29^. The PDMS surfaces were characterized by randomly scanning an area of 10 µm × 10 µm using an atomic force microscope (AFM, Dimension Icon, Bruker, USA) and surface roughness was measured to be 2.2 nm, 1.6 nm, and 2.7 nm (as shown in Fig. 1), respectively, which indicates that the PDMS surfaces could all be regarded as smooth. The thickness of all PDMS films was measured to be 50 ± 2 μm. SDS (Chemical Reagent, Aladdin, ≥ 99.0% (GC), China) powder was dissolved in deionized water to obtain SDS solutions, which was mixed with KCl solution and deionized water for preparing mixture solutions. In the mixture solutions, the concentration of KCl was fixed at 1 mM and that of SDS was 0, 0.12, 0.82, 1.64, 2.46, 4.92, 6.56, 8.2 mM, respectively. All solutions were stored in clean glass containers, which were washed with anhydrous ethanol, acetone and deionized water in advance. All mixture droplets were used within 24 h. For simplicity, the initial KCl concentration of mixture solutions and droplets was fixed at 1 mM in the latter part if not otherwise prescribed. As reported in Ref.^27^, the existence of electrolyte such as KCl and NaCl has an influence on the formation of micelle and in our experiments, 1 CMC for dilute SDS droplets containing 1 mM KCl is about 7.1 ± 0.6 mM. Surface tension of dilute SDS solutions was measured using a dynamic contact angle measuring devices and tensiometer (DCAT11, Dataphysics, Germany). Before the measurement, the sample containers were washed consecutively with acetone, anhydrous ethanol and deionized water, respectively, and then the containers were put in the dryer to avoid impurities that would affect the surface tension measurement results, and then an appropriate amount of mixture aqueous solution was carefully poured into the sample containers. A PT 11 Wilhelmy plate with the width of 19.9 mm and the thickness of 0.2 mm was washed, then burned red with an alcohol lamp to remove impurities, and the Wilhelmy plate was left to cool completely before being placed in the instrument and setting the measurement parameters. Then the plate was inserted into the mixture solutions for measuring the surface tension of the mixture solutions. The ambient temperature and relative humidity were 20 ± 1 °C and 36 ± 2%, respectively. Each experiment was conducted at least three times to ensure experimental reproducibility.

**Figure 2:**
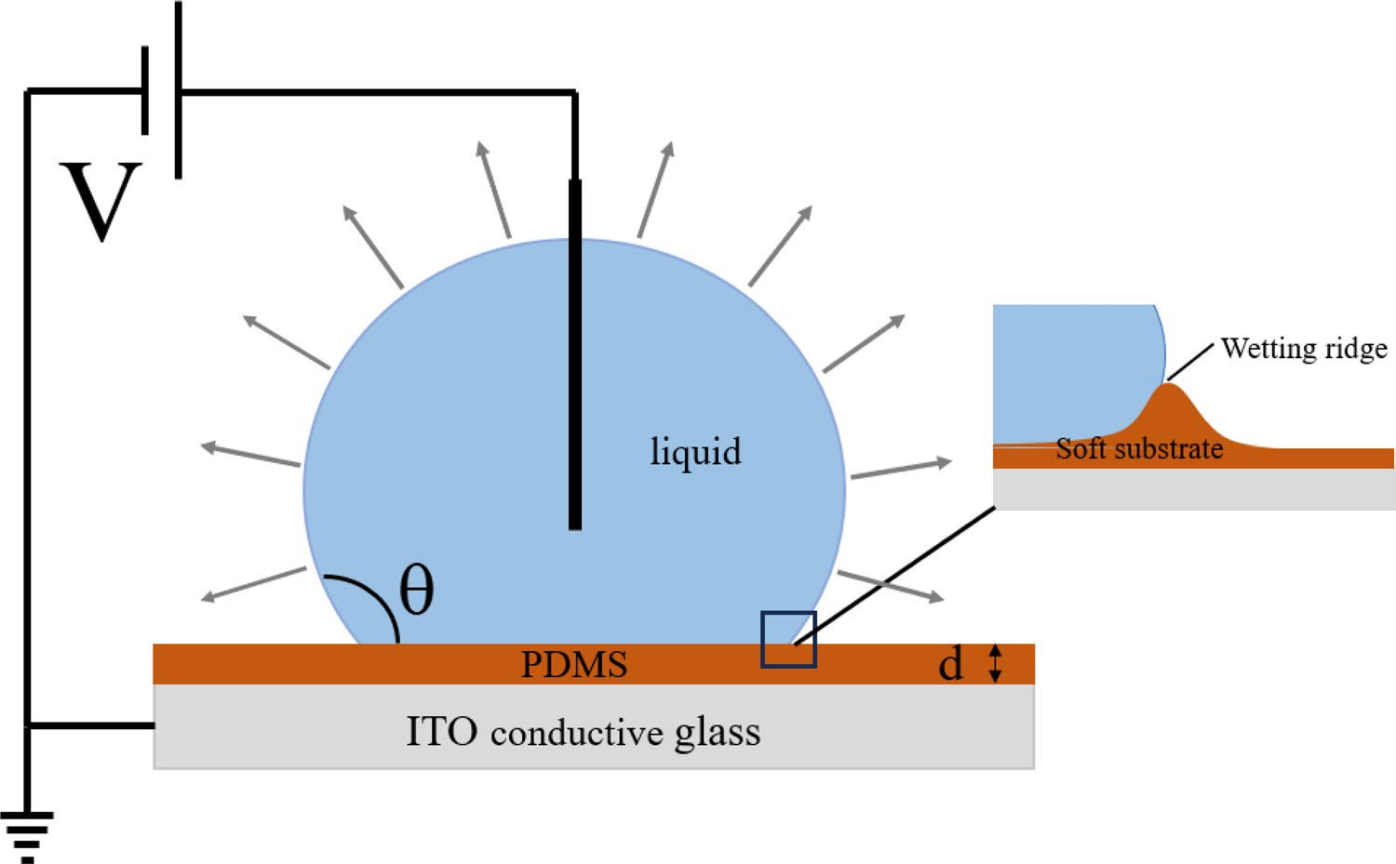
Schematic of experimental setup.

Figure 2 shows the experimental setup of EW on planar PDMS surfaces. Mixture solutions with about 2.0 μL was gently deposited on these surfaces using an NE 30 needle, then a 0.1 mm-diameter platinum wire was inserted into the center of the droplet without touching the PDMS surfaces, and finally a DC voltage was applied to the system using a power amplifier (677B, TREK, USA) at an increasing rate of 10 V/s. A droplet shape analyzer (DSA 30, Krüss, Germany) was made available as soon as possible to record the experimental process at 1 frame per second (fps). Binding the needle with the platinum wire, CAH for dilute SDS droplets containing small amount of KCl on planar PDMS surfaces with the presence of a DC electric field was obtained using the injection/extraction method. The ambient temperature and relative humidity for EWOD experiments were 22 ± 1 °C and 36 ± 2%, respectively. Each experiment was repeated at least thrice to ensure experimental reproducibility.

Evaporation of sessile dilute SDS droplets with about 2.0 μL on planar PDMS surfaces when a DC electric field was applied was also studied using the DSA30 with a recording speed of 1 fps, as shown in Fig. 2. Initial SDS concentration ($c_{{{\text{SDS}}}}^{i}$) was 0 mM, 0.82 mM, 2.46 mM and 4.92 mM, respectively. The magnitude of applied DC voltage was 0 V, 40 V, 200 V, and 250 V, respectively. The experimental temperature and relative humidity were 19 ± 1 °C and 36 ± 2%, respectively. Each experiment was performed thrice to ensure the experimental reproducibility.

## Results and discussion

### EW on PDMS surfaces

**Figure 3:**
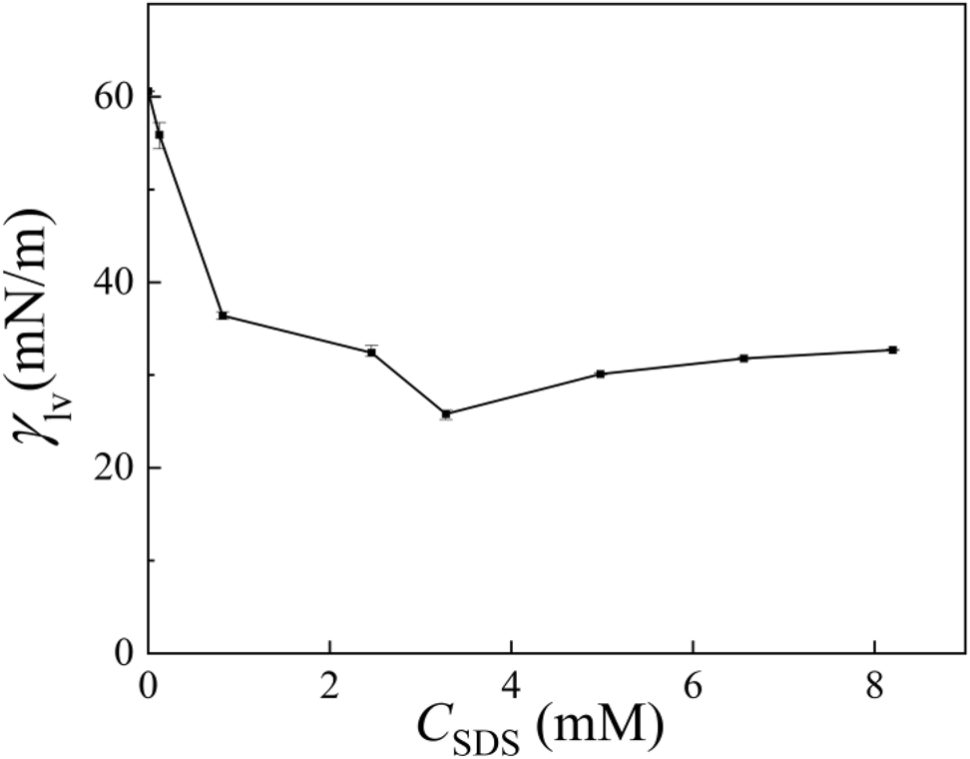
Surface tension of dilute SDS solutions containing 1 mM KCl.

Figure 3 shows the values of surface tension for dilute SDS solutions containing 1 mM KCl. At first, the surface tension decreased greatly with continuous addition of SDS molecules until the concentration of SDS was about 3.74 mM, at which the surface tension had a minimum. The impurity in SDS powder might be responsible for the existence of such a minimum^30^. Then the surface tension increased slowly and kept nearly unchanged until the SDS concentration approached to 1 CMC.

**Figure 4:**
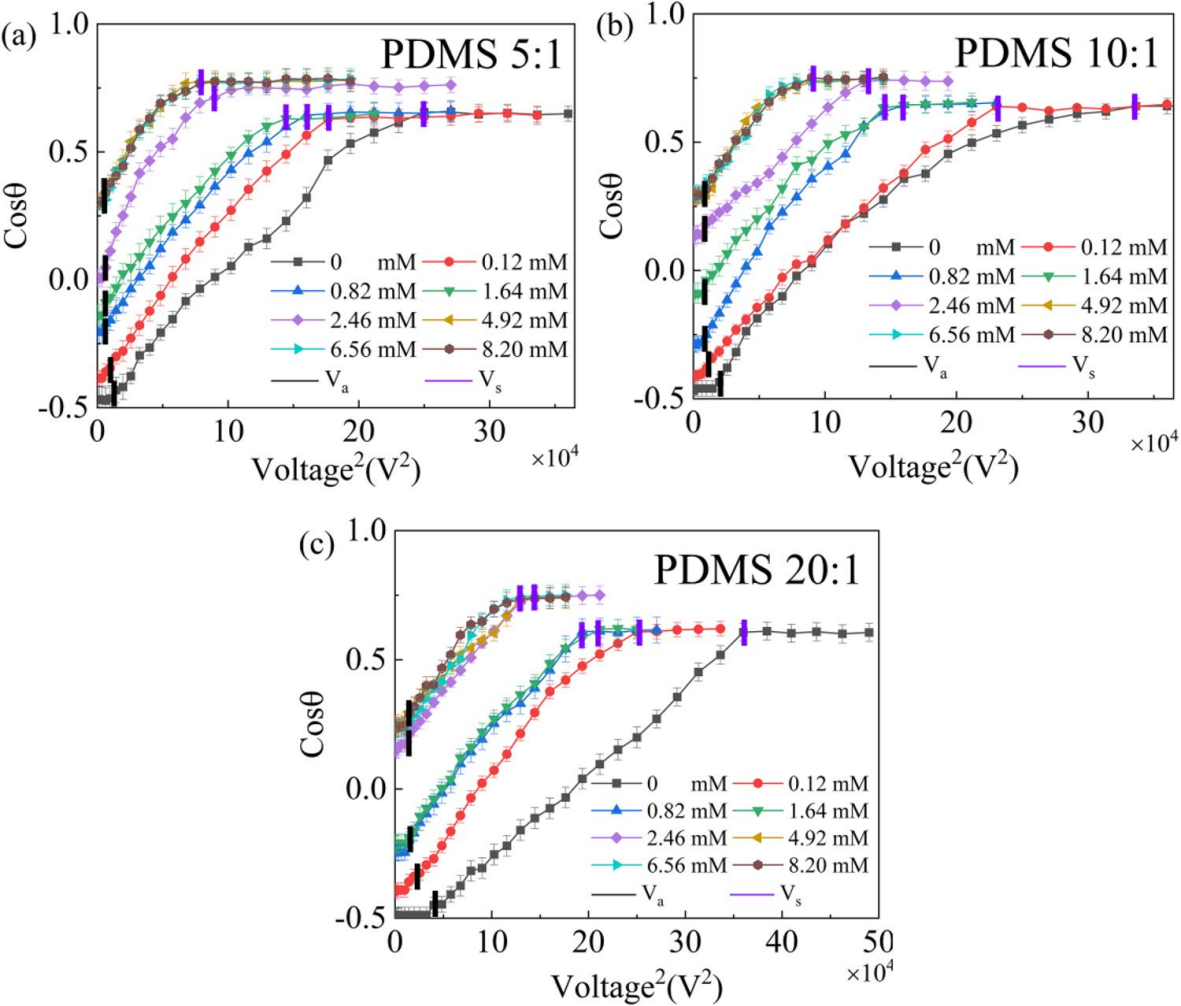
Curves of the cosine of apparent contact angle of dilute SDS droplets on different PDMS films versus the square of applied voltage. (**a**) PDMS 5:1, (**b**) PDMS 10:1, (**c**) PDMS 20:1.

**Figure 5:**
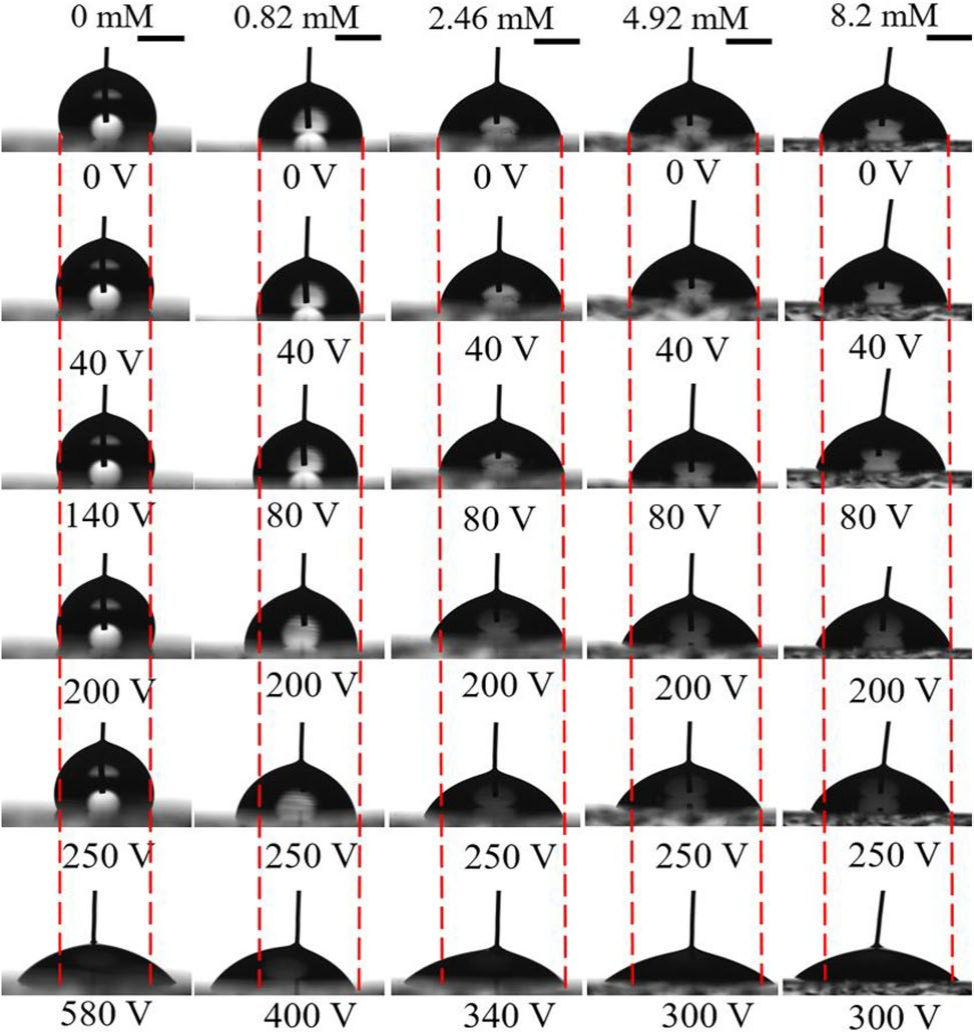
Snapshots of SDS aqueous droplets on the PDMS 10:1 surface under a DC field. All inserted scale bars represent 1 mm.

The cosine value of apparent contact angle for dilute SDS droplets versus the square of applied voltage was given in Fig. 4. Two characteristic values of voltage were found. The first one is actuation voltage $V_{{\text{a}}}$, which has been widely accepted to be related to CAH^31^. The second one is saturation voltage $V_{{\text{s}}}$. The existence of $V_{{\text{s}}}$ might be attributed to charge trapping^24^ and it should be studied further. When the applied voltage is great than $V_{{\text{s}}}$, the instantaneous contact angle no longer decreases and such a contact angle is called as saturation contact angle $\theta_{{\text{s}}}$. Though a lot of researchers have investigated the phenomenon of contact angle saturation, however up till now there is no unique explanation of its origin^32,33^. As an example, the snapshots of SDS aqueous droplets on the PDMS 10:1 surface under different voltages were given in Fig. 5. Table 1 summarizes the values of $V_{{\text{a}}}$, $V_{{\text{s}}}$ and $\theta_{{\text{s}}}$. From Table 1, it was found that $V_{{\text{a}}}$ increased with increasing mass ratio of PDMS surfaces when SDS concentration was fixed. Such a phenomenon could be attributed to the additional contribution of substrate deformation induced by $\gamma_{{{\text{lv}}}}^{{}} \sin \theta$ to CAH^34,35^. And a larger $V_{{\text{s}}}$ was found in the electrowetting of SDS aqueous droplets on a PDMS surface with a higher mass ratio. The reason is still unclear and it should be studied further. However, there was very small difference in $\theta_{{\text{s}}}$ for dilute SDS droplets with the same SDS concentration wetting on PDMS surfaces with different mass ratios. For a given PDMS surface, SDS concentration was also found to have an obvious influence on the wetting characteristics of dilute SDS droplets when a DC electric field was present. $V_{{\text{a}}}$ was found to decrease with the increase of SDS concentration when it was no more than 0.82 mM, above which $V_{{\text{a}}}$ ceased to decrease. $V_{{\text{s}}}$ decreased with increasing SDS concentration when it ranging from 0 mM to about 4.92 mM, above which $V_{{\text{s}}}$ no longer decreased. $\theta_{{\text{s}}}$ was found to decrease from about 50° for the case of KCl aqueous droplets and SDS aqueous droplets with a low SDS concentration to about 40° with more introduction of SDS molecules into the liquid.

**Table 1:** Experimental data of actuation voltage, saturation voltage and saturation contact angle.

$c_{{\text{i}}}$(mM)	$V_{{\text{a}}}$(V)			$V_{{\text{s}}}$(V)			$\theta_{{\text{s}}}$		
	PDMS 5:1	PDMS 10:1	PDMS 20:1	PDMS 5:1	PDMS 10:1	PDMS 20:1	PDMS 5:1	PDMS 10:1	PDMS 20:1
0	100	140	220	500	580	640	50° ± 1°	50° ± 1°	52° ± 1°
0.12	80	100	120	400	460	500	50° ± 1°	50° ± 1°	50° ± 1°
0.82	60	80	100	380	400	440	49° ± 2°	49° ± 1°	52° ± 1°
1.64	60	80	100	360	380	420	50° ± 1°	51° ± 1°	51° ± 1°
2.46	60	80	100	340	360	380	41° ± 1°	42° ± 2°	41° ± 1°
4.92	60	80	100	280	300	360	39° ± 1°	42° ± 1°	41° ± 1°
6.56	60	80	100	280	300	360	39° ± 1°	42° ± 1°	42° ± 1°
8.20	60	80	100	280	300	360	39° ± 1°	42° ± 1°	42° ± 1°

### Evaporation of dilute SDS droplets on planar PDMS surfaces under a DC electric field

**Figure 6:**
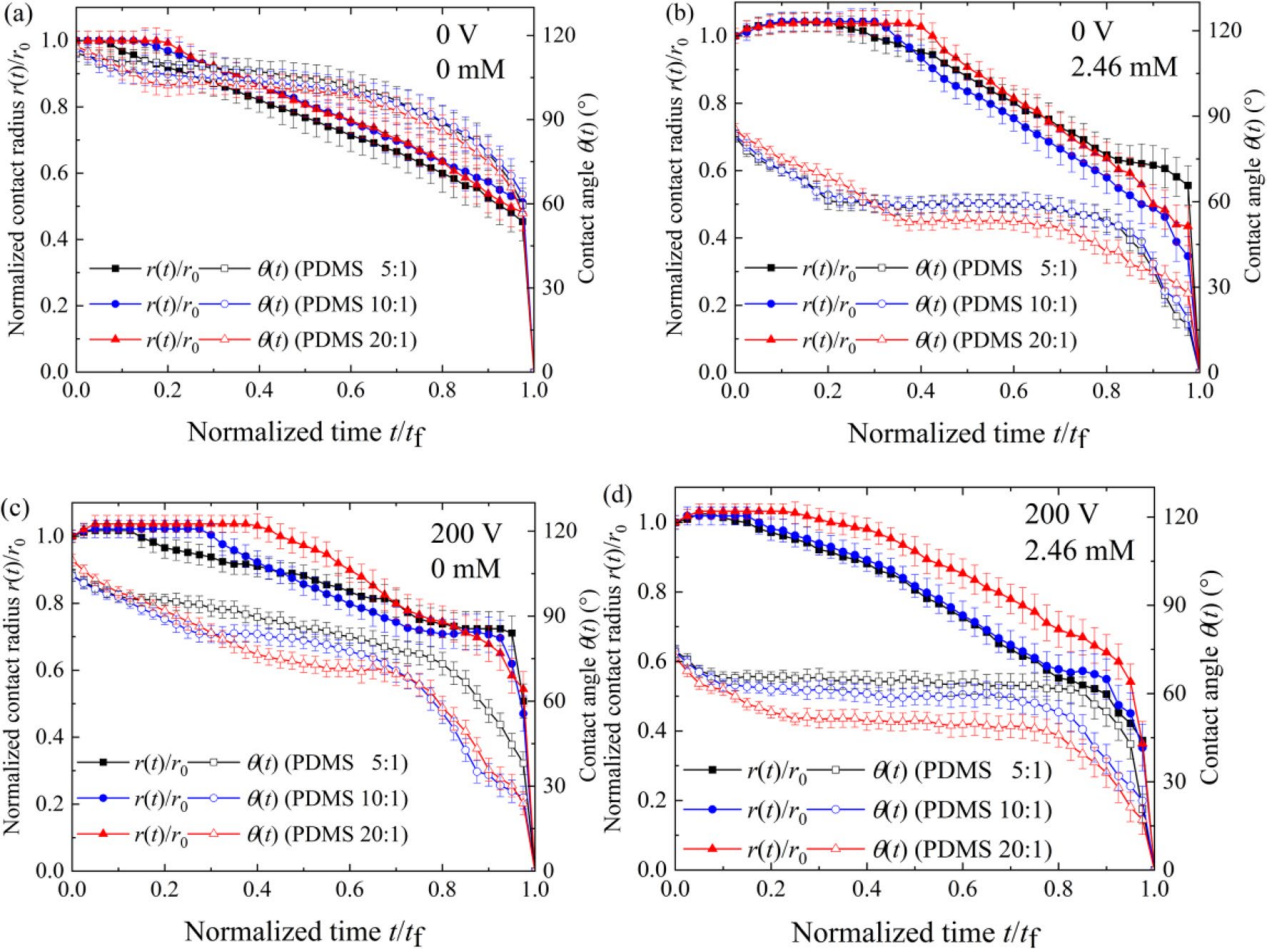
Evaporation curves of dilute SDS droplets on different PDMS surfaces. (**a**) $c_{{{\text{SDS}}}}^{i} = 0{\text{ mM}}$ and $V = 0\;{\text{V}}$, (**b**) $c_{{{\text{SDS}}}}^{i} = 2.46{\text{ mM}}$ and $V = 0\;{\text{V}}$, (**c**) $c_{{{\text{SDS}}}}^{i} = 0\;{\text{mM}}$ and $V = 200\;{\text{V}}$, (**d**) $c_{{{\text{SDS}}}}^{i} = 2.46\;{\text{mM}}$ and $V = 200\;{\text{V}}$.

Figure 6 shows the evaporation curves of normalized contact radius—*r*(*t*)/* r*_0_ (where *r*(*t*) and *r*_0_ are the instantaneous contact radius and the initial contact radius, respectively) and contact angle *θ*(t) versus normalized time –*t/t*_f_ (where *t*_f_ is the total evaporation time) for dilute SDS droplets on planar PDMS surfaces at different applied voltage, respectively. When no electric field was present, the evaporation of 1 mM KCl droplets started with the CCR mode, as shown in Fig. 6a. With minor addition of SDS molecules, the sessile droplets experienced a short-period stage of spontaneous spreading at the beginning of evaporation when no voltage is applied (Fig. 6b), and this phenomenon can be attributed to surfactant molecules transferring from the droplet to the PDMS surface^36^. A short-time spontaneous spreading was also found at the early stage of the evaporation of 1 mM KCl droplets under a DC voltage of 200 V (as shown in Fig. 6c). This may be because at the contact line the Maxwell stress broke the balance of forces, and there was an outward resultant force^37,38^. For the case of dilute SDS droplets with $c_{{{\text{SDS}}}}^{i}$ of 2.46 mM at 200 V (Fig. 6d), the duration of CCR stage was much shorter than those for the same droplet on the same substrate without the presence of an electric field (the presence of an electric field exerts Maxwell force to weaken CAH) and the droplets without the addition of SDS molecules on the same substrate under the same electric field (the introduction of SDS molecules greatly reduce the surface tension of the liquid and thus there will be a less elastic stored energy which acts as an additional contribution to CAH).

From Fig. 6, it was found that substrate elasticity obviously influenced the evaporation characteristics, which could be attributed to substrate deformation induced by $\gamma_{{{\text{lv}}}}^{{}} \sin \theta$^34,35^. Under the action of $\gamma_{{{\text{lv}}}}^{{}} \sin \theta$, the PDMS surface is deformed and the elastic stored energy per unit length of contact line is of the order of $r_{lv}^{2} \sin^{2} \theta (1 - \upsilon^{2} )/E$^31,32^, where $E$ and $\upsilon$ being the Young's modulus and Poisson's ratio of the PDMS surface, respectively. Previous studies have demonstrated that the PDMS film with a higher mass ratio usually has a lower Young’s modulus^35^. Thus more elastic energy is stored in the substrate with a higher mass ratio. For such an energy serving as a barrier to prevent the contact line from moving, there will be a longer CCR stage for the case of evaporation of dilute SDS droplets on a planar PDMS surface with a higher mass ratio.

**Figure 7:**
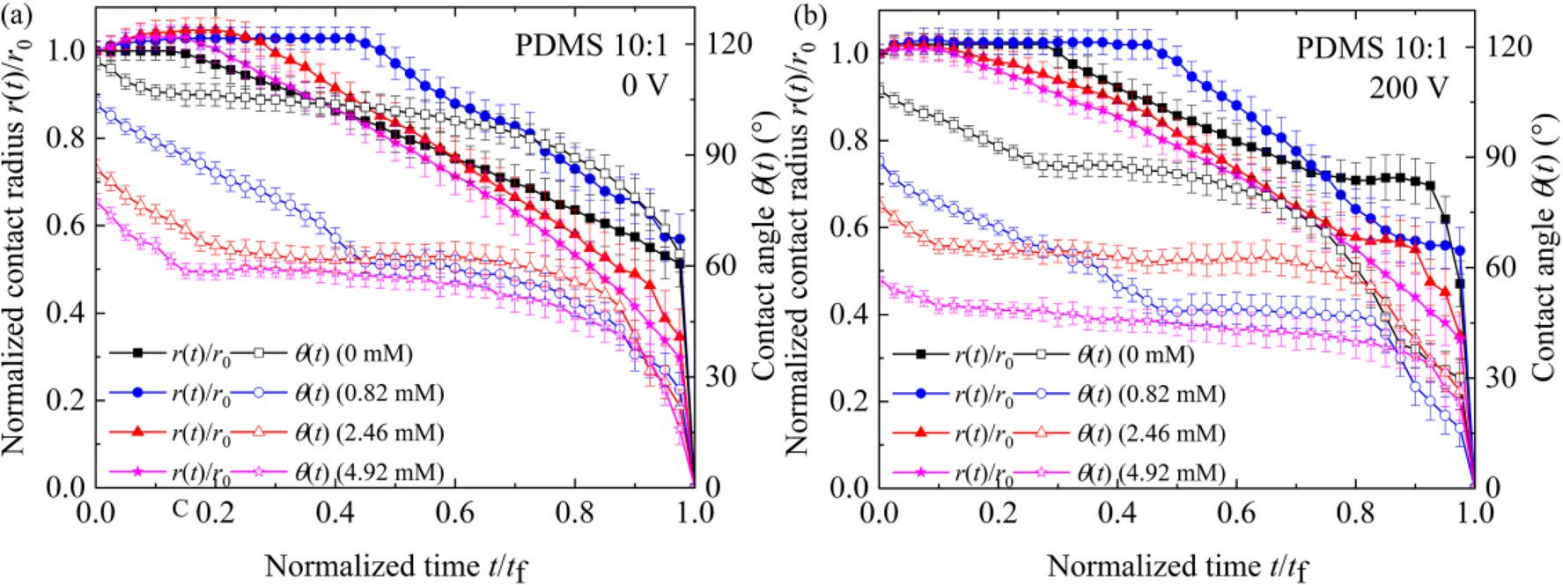
Evaporation curves of dilute SDS droplets on planar PDMS surfaces under different voltages. (**a**) 0 V, (**b**) 200 V.

**Figure 8:**
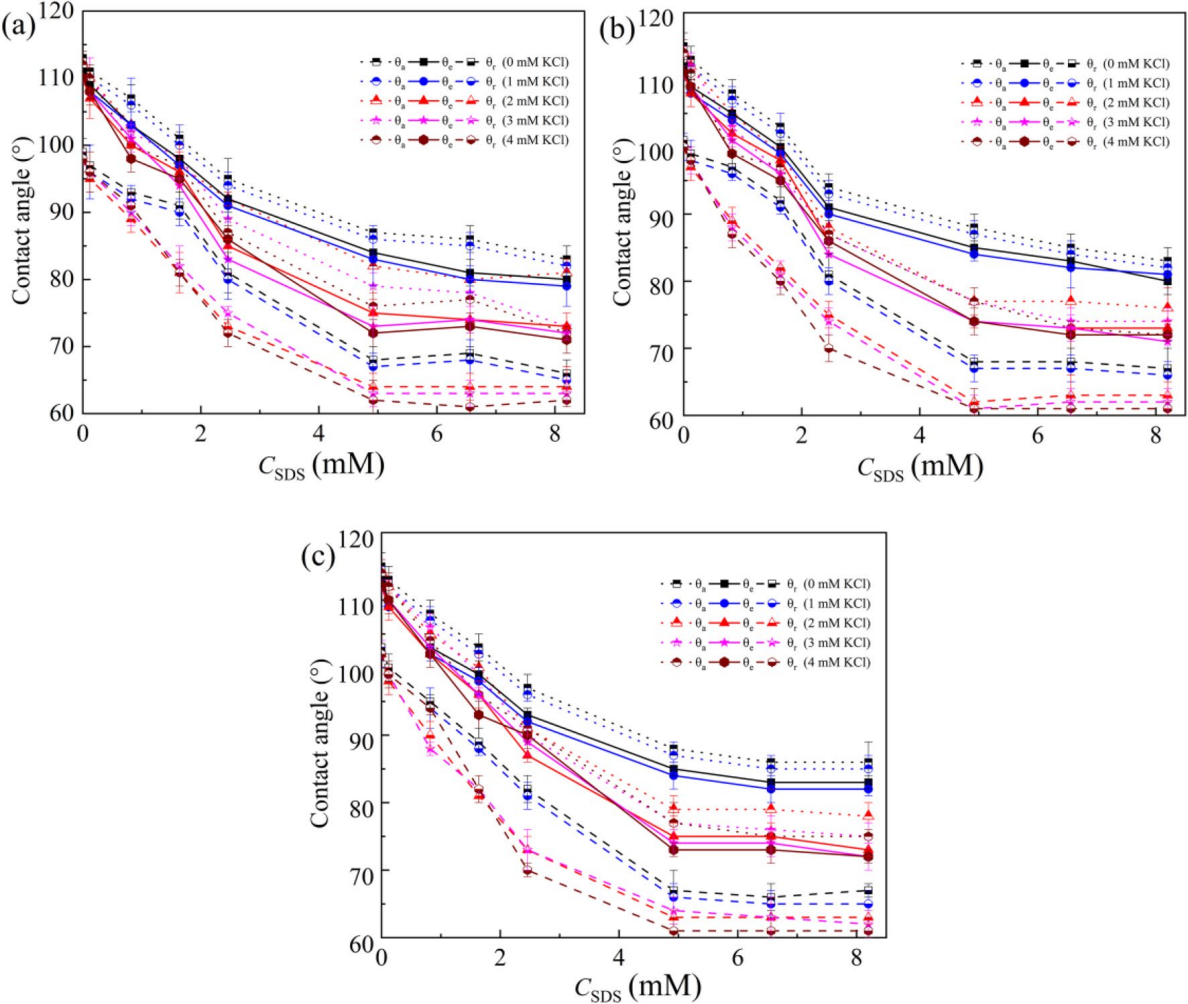
CAH of dilute SDS droplets on PDMS surfaces. (**a**) PDMS 5:1, (**b**) PDMS 10:1, (**c**) PDMS 20:1.

To elucidate the influence of SDS concentration on evaporation characteristics of SDS aqueous droplets under a DC electric field, the curves for dilute SDS droplets with $c_{{{\text{SDS}}}}^{i}$ of 0, 0.82, 2.46 and 4.92 mM evaporating on a planar PDMS 10:1 surface at 0 V and 200 V were shown in Fig. 7a,b, respectively. A short-period stage of spontaneous spreading was also observed at the beginning of the evaporation of sessile dilute SDS droplets, which could be attributed to the transfer of SDS molecules from the liquid–vapor surface near the contact line to the solid–vapor interface^36^. Moreover, SDS concentration was found to have an obvious influence on the duration of CCR stage and there was a longest CCR stage for the case of dilute SDS droplets with $c_{{{\text{SDS}}}}^{i}$ of 0.82 mM. To elucidate the influence of SDS concentration on CCR duration, advancing ($\theta_{{\text{a}}}$), apparent ($\theta_{{\text{e}}}$) and receding ($\theta_{{\text{r}}}$) contact angles for dilute SDS droplets containing a small amount of KCl was measured^39,40^, as shown in Fig. 8. From Fig. 8, it is easily found that KCl concentration had only a very small influence on CAH when no SDS molecules was introduced into the liquid. Thus, the duration of CCR stage for KCl aqueous droplets was relatively short. However, when SDS molecules were introduced, both $\theta_{{\text{a}}}$ and $\theta_{{\text{r}}}$ decreased with increasing KCl concentration and increasing SDS concentration when SDS concentration was less than 4.92 mM, and $\theta_{{\text{r}}}$ decrease more greatly. When dilute SDS droplets evaporates on a PDMS surface, both the instantaneous SDS concentration and KCl concentration increase with the loss of water, resulting in the variation of $\theta_{{\text{a}}}$ and $\theta_{{\text{r}}}$, and therefore that of the pinning force $\gamma_{{{\text{lv}}}} \left( {\theta_{{\text{r}}} - \theta_{{\text{a}}} } \right)$. For dilute SDS droplets with a very low initial SDS concentration, $\theta_{{\text{r}}}$ decreases more greatly than $\theta_{{\text{a}}}$ with the loss of water, leading to a larger dimensionless pinning force $\left( {\theta_{{\text{r}}} - \theta_{{\text{a}}} } \right)$. Therefore, longest CCR stage was observed during the evaporation of dilute SDS droplets with $c_{{{\text{SDS}}}}^{i}$ of 0.82 mM and the duration of CCR stage for dilute SDS droplets with $c_{{{\text{SDS}}}}^{i}$ of 4.92 mM was shorter than that for dilute SDS droplets with $c_{{{\text{SDS}}}}^{i}$ of 2.46 mM.

**Figure 9:**
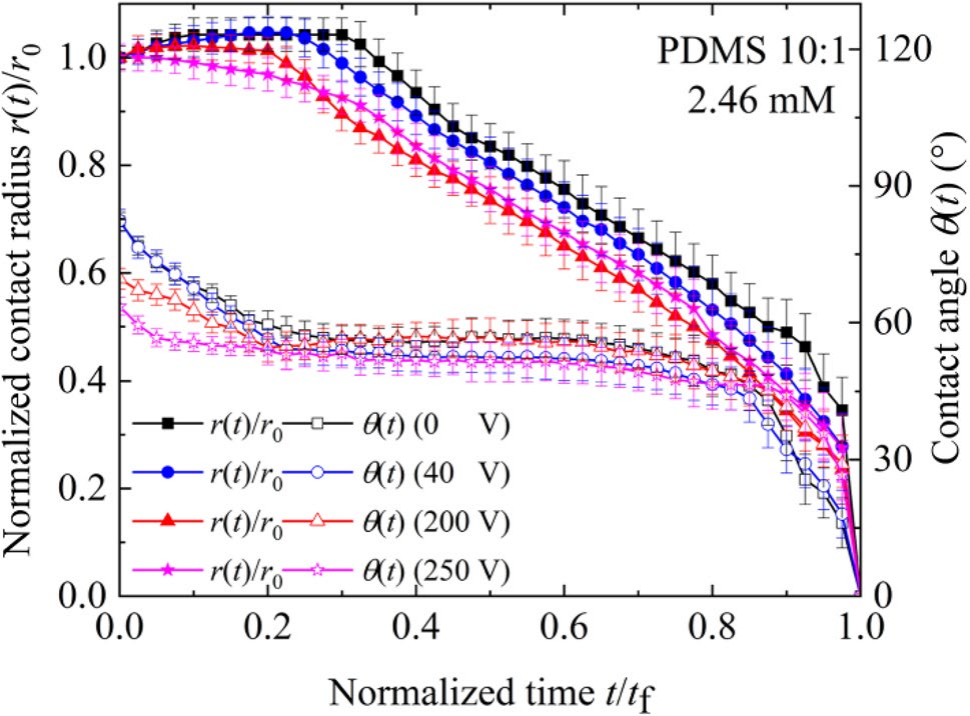
Evaporation curves of dilute SDS droplets with an initial SDS concentration of 2.46 mM on different PDMS surfaces.

**Figure 10:**
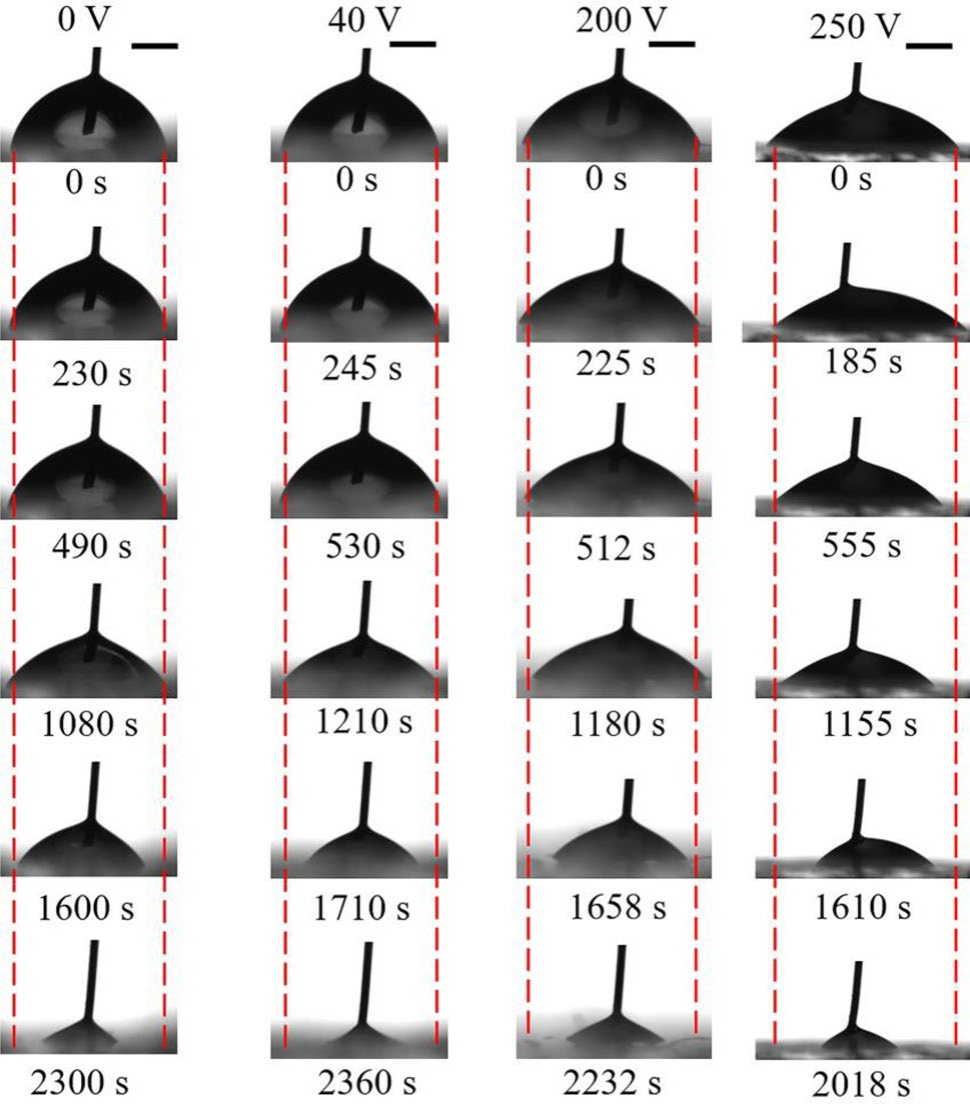
Snapshots of SDS aqueous droplets with $c_{{{\text{SDS}}}}^{i}$ of 2.46 mM evaporating on PDMS 10:1 under different DC voltages. All inserted scale bars represent 1 mm.

**Figure 11:**
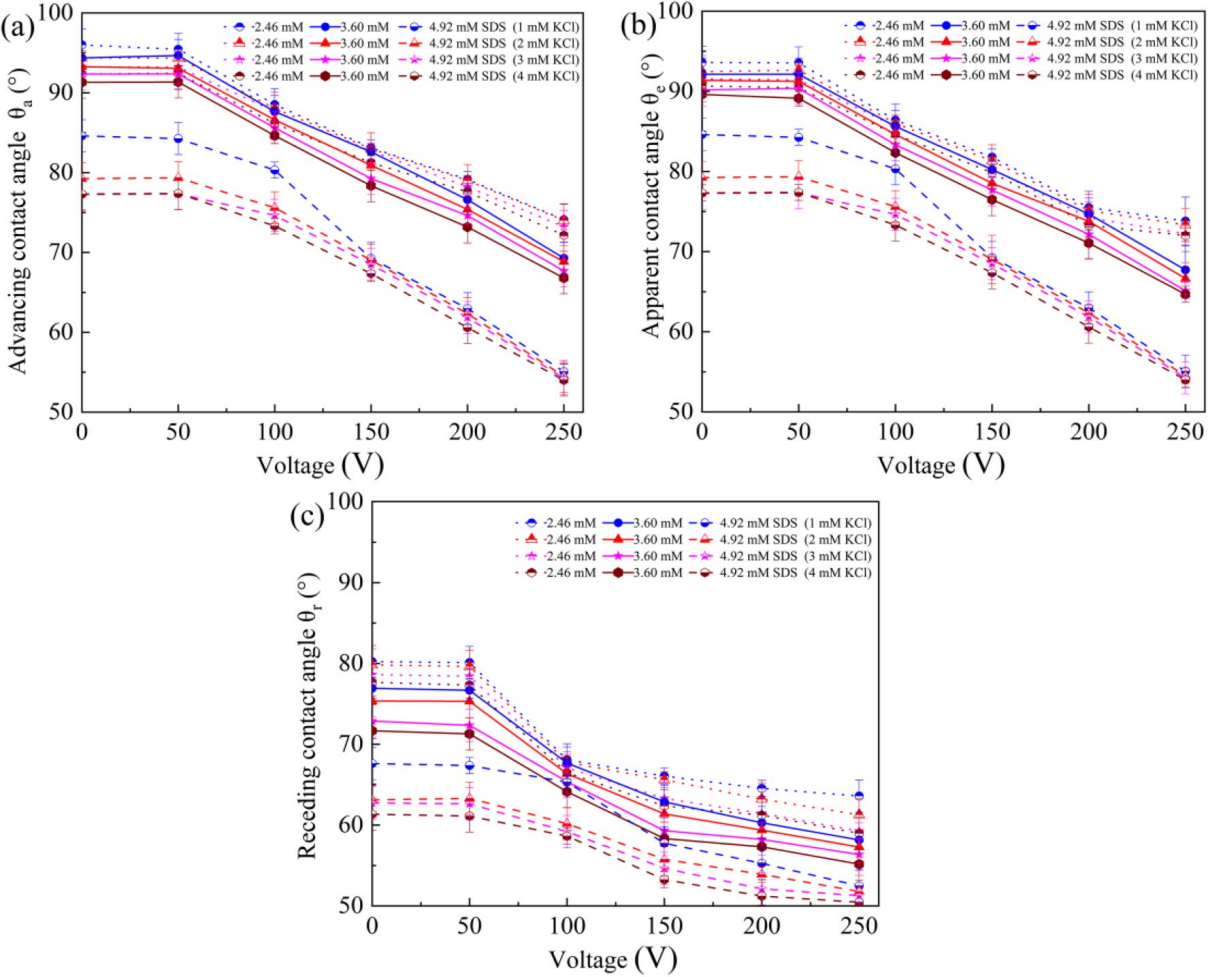
Contact angles for dilute SDS droplets on a PDMS 10:1 surface under a DC electric field. (**a**) advancing contact, (**b**) apparent contact angle, (**c**) receding contact angle.

To demonstrate the influence of applied voltage on evaporation characteristics of sessile SDS aqueous droplets, the curves for dilute SDS droplets with $c_{{{\text{SDS}}}}^{i}$ of 2.46 mM evaporating on a planar PDMS 10:1 surface under different DC voltages were given in Fig. 9. The snapshots of evaporating SDS aqueous droplets on the PDMS 10:1 surface under different voltages were listed in Fig. 10. At 0 V or 40 V, there was still a relatively long CCR stage. However, the duration of CCR stage for 200 V was much shorter and a shortest CCR stage was observed for 250 V. For CCR stage being attributed to CAH, the experiment curves shown in Fig. 9 indicates the magnitude of applied voltage obviously influenced CAH. As water evaporates, the instantaneous SDS concentration and KCl concentration both increase gradually. Therefore, $\theta_{{\text{a}}}$, $\theta_{{\text{e}}}$ and $\theta_{{\text{r}}}$ for dilute SDS droplets under different applied voltage were measured, as shown in Fig. 11. From Fig. 11, it is easily found that the magnitude of applied DC voltage has an obvious influence on CAH. SDS concentration and KCl concentration also influence CAH.

**Figure 12:**
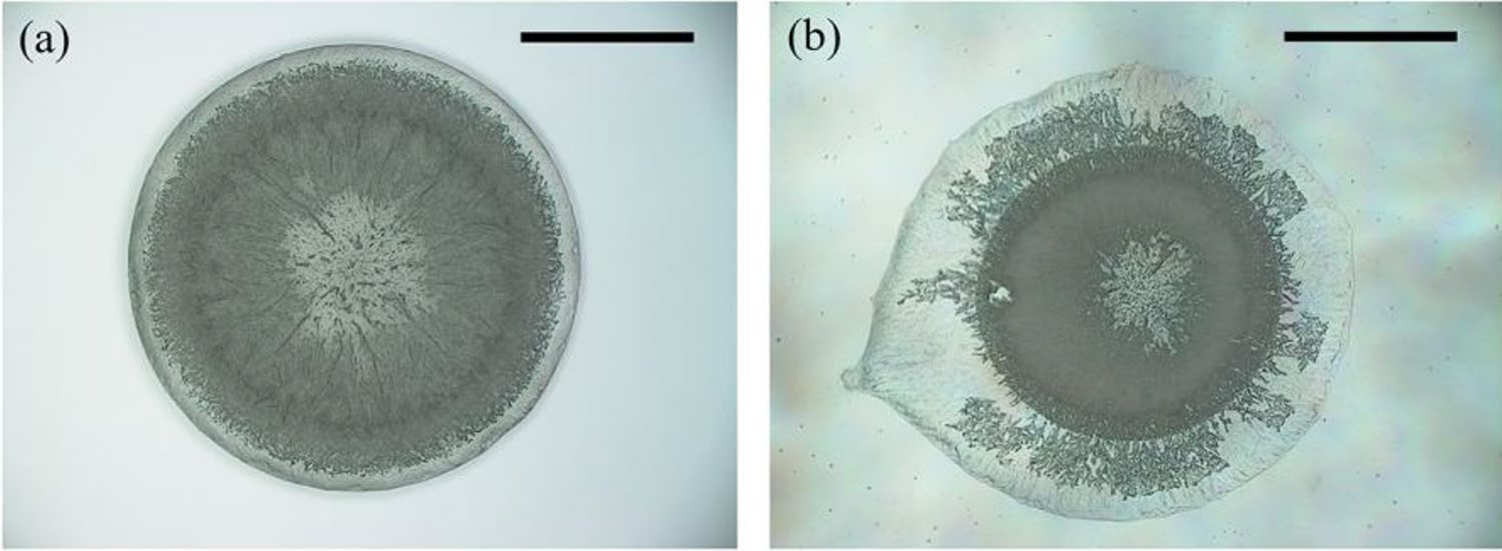
Evaporative deposition of dilute SDS droplets with an initial SDS concentration of 2.46 mM under different DC voltages. (**a**) *V* = 0 V, (**b**) *V* = 200 V. All inserted scale bars represent 1 mm.

Figures 12a,b show the evaporative deposition of dilute SDS droplets with $c_{{{\text{SDS}}}}^{i}$ of 2.46 mM at 0 V and 200 V using a KH-8700 microscope (Hirox, Japan), respectively. From Fig. 10, it is easily concluded that electric field had an obvious influence on evaporative deposition and it should be studied further.

## Conclusion

Wettability and evaporation of dilute SDS droplets on planar PDMS surfaces with different mass ratios with the presence of a DC electric field were experimentally investigated. It was found that substrate elasticity has an obvious influence on actuation voltage, which could be attributed to the additional contribution of substrate deformation under the action of $\gamma_{{{\text{lv}}}}^{{}} \sin \theta$ to CAH. Substrate elasticity also influences the saturation voltage. The magnitude of applied voltage obviously influenced CAH for dilute SDS droplets on planar PDMS surfaces and therefore influenced the evaporation characteristics of sessile dilute SDS droplets under a DC electric field. Substrate elasticity and SDS concentration were found to also have an influence on evaporation characteristics of dilute SDS droplets. We envisioned this work may be helpful for widening the application of surfactants and electric field in micro-/nano-fluidics and self-assembly of micro-/nano-particles, etc.

## Data Availability

The data supporting the findings of this study are available from the corresponding author upon reasonable request.
